# Preparation and Evaluation of Dexamethasone (DEX)/Growth and Differentiation Factor-5 (GDF-5) Surface-Modified Titanium Using β-Cyclodextrin-Conjugated Heparin (CD-Hep) for Enhanced Osteogenic Activity In Vitro and In Vivo

**DOI:** 10.3390/ijms18081695

**Published:** 2017-08-03

**Authors:** Dae Hyeok Yang, Sun-Jung Yoon, Deok-Won Lee

**Affiliations:** 1 Institute of Cell and Tissue Engineering, College of Medicine, The Catholic University of Korea, Seoul 06591, Korea; yangdh@catholic.ac.kr; 2 Department of Orthopedic Surgery, Research Institute of Clinical Medicine of Chonbuk National University, Biomedical Research Institute of Chonbuk National University Hospital, Jeonju 54907, Korea; sjyoon_kos@naver.com; 3 Department of Oral & Maxillofacial Surgery, Kyung Hee University Dental Hospital at Gangdong, Kyung Hee University, Seoul 05278, Korea

**Keywords:** growth and differentiation factor-5, dexamethasone, β-cyclodextrin, heparin, bone formation, osseointegration

## Abstract

The most ideal implant models in the dental and orthopedic fields to minimize the failure rate of implantation involve the improvement of osseointegration with host bone. Therefore, a focus of this study is the preparation of surface-modified titanium (Ti) samples of disc and screw types using dexamethasone (DEX) and/or growth and differentiation factor-5 (GDF-5), as well as the evaluation of their efficacies on bone formation in vitro and in vivo. X-ray photoelectron spectroscopy (XPS), scanning electron microscopy (SEM) and contact angle measurement were used to evaluate the surface chemical composition, surface morphology and wettability, respectively. The results showed that implant surfaces were successfully modified with DEX and/or GDF-5, and had rough surfaces along with hydrophilicity. DEX, GDF-5 or DEX/GDF-5 on the surface-modified samples were rapidly released within one day and released for 28 days in a sustained manner. The proliferation and bone formation of MC3T3-E1 cells cultured on pristine and surface-modified implants in vitro were examined by cell counting kit-8 (CCK-8) assay, as well as the measurements of alkaline phosphatase (ALP) activity and calcium deposition, respectively. MC3T3-E1 cells cultured on DEX/GDF-5–Ti showed noticeable ALP activity and calcium deposition in vitro. Active bone formation and strong osseointegration occurred at the interface between DEX/GDF-5–Ti and host bone, as evaluated by micro computed-tomography (micro CT) analysis. Surface modification using DEX/GDF-5 could be a good method for advanced implants for orthopaedic and dental applications.

## 1. Introduction

Titanium (Ti) and its alloys have been widely used as implants in the orthopedic and dental fields due to their good mechanical properties and biocompatibility [1]. However, clinicians need advanced Ti implants because the implantation often fails by causing unsatisfactory osseointegration at the interface between the implants and host bone. To date, several physical and chemical surface modification methods, such as machining, grinding, polishing, blasting, acid-etching, hydrogen peroxide (H_2_O_2_)-etching, alkali-etching, sol-gel coating, anodic oxidation, and chemical vapor deposition and coating, have been developed for improving osseointegration [2,3,4]. Nevertheless, such surface modifications methods still seem to be unsatisfactory to clinicians.

Recently, biological surface modification methods using bone-forming drugs or growth factors have been attempted to improve the osseointegration at the interface between implants and host bone. Here, dexamethasone acetate (DEX) and growth and differentiation factor-5 (GDF-5) were used as biological modifiers. DEX and GDF-5 could be attractive dual materials to enhance osseointegration [5,6]. DEX induces osteogenic differentiation in vitro and increases alkaline phosphatase (ALP) activity, the expression of osteocalcin (OC) and bone sialoprotein (BSP) levels [5]. GDF-5 is a remarkable factor for bone tissue engineering because it can improve both osteogenesis and angiogenesis [6]. Therefore, the combination of DEX and GDF-5 has the potential to promote biological surface modifications to improve the osseointegration of Ti implants.

β-Cyclodextrin (β-CD) and heparin enable complex formation of DEX and GDF-5, respectively [7,8]. β-CD forms an inclusion complex with DEX [7], and it has a heparin binding site [8]. Therefore, DEX and GDF-5 might be amenable to coating onto metals that have been chemically surface-treated with β-CD-conjugated heparin (CD–Hep). The primary hydroxyl group of β-CD can be substituted with an amine group [9], allowing for the conjugation with the carboxyl group of heparin through amide bond formation. This surface modification has not yet been reported, and would be beneficial for dental and orthopaedic implants.

In the present study, DEX/GDF-5 surface-modified Ti disc and screw samples were prepared, followed by the investigation of their ability to enhance bone formation in vitro and in vivo, as well as osseointegration in vivo. The impact of surface-modified Ti disc samples on MC3T3-E1 cell function was investigated in a series of in vitro tests, including cell proliferation evaluation, alkaline phosphatase (ALP) activity and calcium deposition. The expression of mRNA genes including type I collagen (COL I), alkaline phosphatase (ALP) and osteocalcin (OCN), was also evaluated. The in vivo animal tests including micro-computed tomography (micro-CT), removal torque and histological evaluations were carried out using a rabbit tibia defect model.

## 2. Results

### 2.1. ^1^H-NMR Spectra of CD–Hep

**Figure 1:**
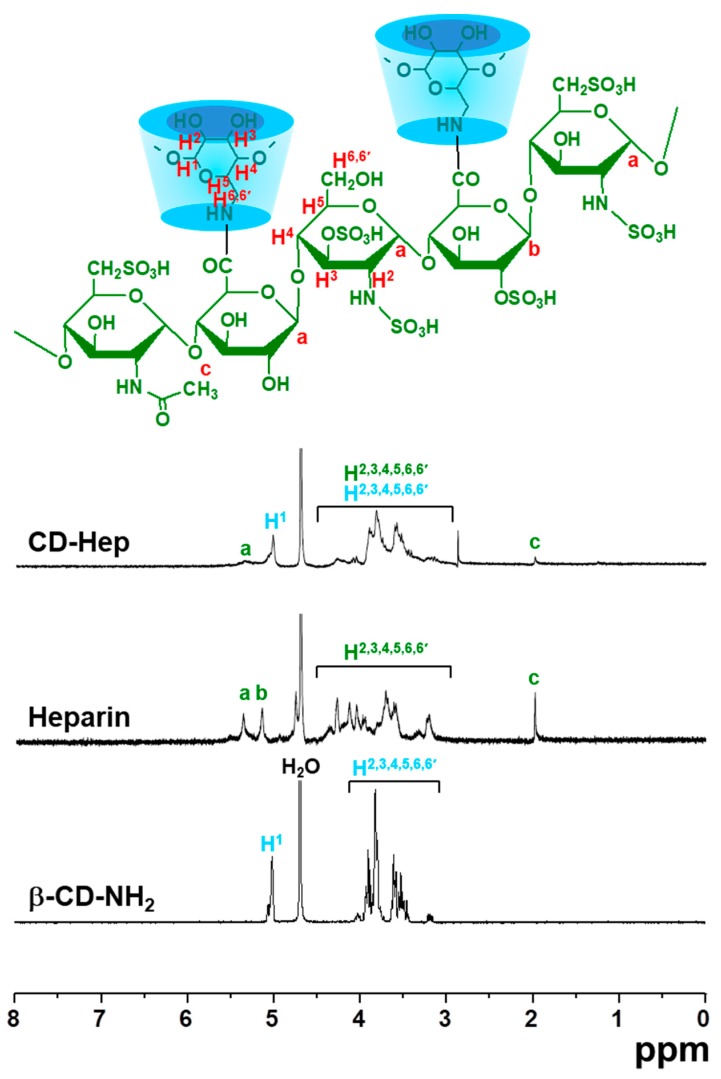
Proton nuclear magnetic resonance (^1^H-NMR) spectra of β-cyclodextrin (β-CD)-conjugated heparin (CD–Hep), measured in D_2_O. The signals of a, b and c indicates the acetyl group of *N*-acetyl-d-glucosamine in the molecular structure of heparin, the H^1^ of 6-monodeoxy-6-monoamino-β-cyclodextrin (β-CD–NH_2_) and the H^1^ of d-glucuronic acid, respectively.

The formation of CD–Hep was confirmed using proton nuclear magnetic resonance (^1^H-NMR) spectroscopy, measured in D_2_O (Figure 1). The peaks at 1.99 ppm, 5.02 ppm and 5.35 ppm were assigned to the acetyl group of *N*-Acetyl-d-glucosamine in the molecular structure of heparin, the H^1^ of 6-monodeoxy-6-monoamino-β-cyclodextrin (β-CD–NH_2_) and the H^1^ of d-glucuronic acid, respectively. This indicated the conjugation of β-CD–NH_2_ to the carboxylic acid group of heparin through amide bond formation. The peaks of H^2,3,4,5,6,6′^ in the molecular structure of heparin and β-CD–NH_2_ were hard to assign because the peaks overlapped.

### 2.2. Surface Chemical Composition

**Figure 2:**
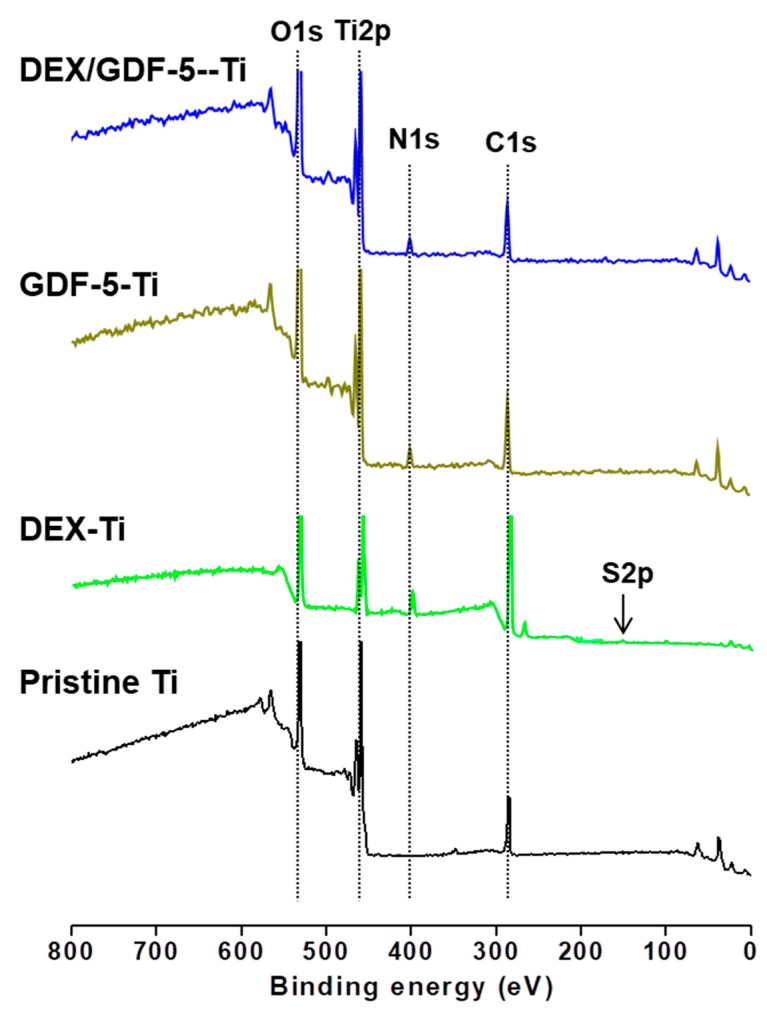
X-ray photoelectron spectroscopy (XPS) spectra of pristine Ti, dexamethasone (DEX)–Ti, growth and differentiation factor-5 (GDF-5)–Ti and DEX/GDF-5–Ti disc samples, as monitored from 100 to 800 eV.

Surface chemical compositions on pristine Ti, and DEX, GDF-5 and DEX/GDF-5 surface-modified Ti samples (DEX–Ti, GDF-5–Ti and DEX/GDF-5–Ti) are presented in Figure 2. Pristine Ti represented typically Ti2p (461.6 eV) and O1s (530.8 eV) XPS signals, because the TiO_2_ layer of pristine Ti was attributed to the O1s signal. The surface contamination of pristine Ti might contribute to the production of C1s. The S2p (162.3 eV) signal was observed on DEX–Ti. This may indicate that CD–Hep was immobilized onto the surface. GDF-5–Ti and DEX/GDF-5–Ti did not show the S2p signal, because GDF-5 fully covered CD–Hep immobilized surface. F1s signal was not observed on DEX–Ti, which indicated that the amount of DEX was below the detection limit. The surface modifications were further explained by examining the percentages of Ti2p, C1s (285.9 eV) and O1s. The percentage of Ti2p on the surface-modified samples was lower than that of pristine Ti. Compared with pristine Ti, the surface-modified samples exhibited an increased percentage of C1s and a decreased percentage of O1s. These results can be ascribed to the surface modification of CD–Hep, DEX and GDF-5.

### 2.3. Surface Morphology

**Figure 3:**
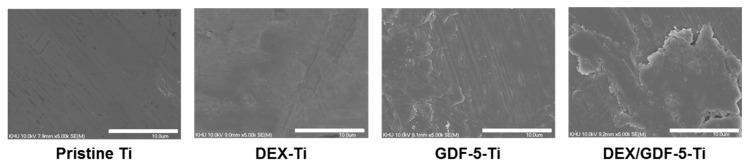
Scanning electron microscopy (SEM) images of pristine Ti, DEX–Ti, GDF-5–Ti and DEX/GDF-5–Ti disc samples observed at a magnification of 5 k. The white bar indicates 10 μm.

Figure 3 shows the surface morphologies of pristine Ti, DEX–Ti, GDF-5–Ti and DEX/GDF-5–Ti samples. Compared with the surface-modified samples, pristine Ti showed a smooth surface morphology. There was no significant difference observed on the surfaces of the surface-modified samples. The results indicated that the rough surfaces of surface-modified Ti samples were produced by the surface modification of DEX, GDF-5 and the combination of both.

### 2.4. Water Contact Angles

Water contact angles on pristine and surface-modified Ti samples are presented in Table 1. The contact angle on pristine Ti sample was 78°. The surface-modified Ti samples showed lower contact angles (47° to 54°) than pristine. DEX–Ti exhibited a higher contact angle than CD–Hep coated Ti (45°) sample due to the hydrophobicity of DEX. The surface modification of DEX–Ti using GDF-5 decreased the contact angle, indicating that hydrophilic GDF-5 was coated onto the surface of the sample.

### 2.5. Coating Efficiency and Release Behavior

**Figure 4:**
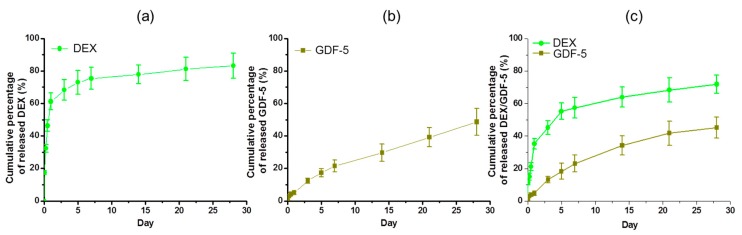
Release behavior of DEX and/or GDF-5 on (**a**) DEX–Ti, (**b**) GDF-5–Ti and (**c**) DEX/GDF-5–Ti disc samples in PBS (pH 7.4) monitored for 28 days. The error bars represent mean ± SD (*n* = 3). This experiment was repeated three times.

The coating efficiencies of DEX, GDF-5 and DEX/GDF-5 on DEX–Ti, GDF-5–Ti and DEX/GDF-5–Ti were 38.5 ± 2.5%, 52.2 ± 3.7% and 39.3 ± 4.1%/51.9 ± 2.8%, respectively. Figure 4 shows the 30-day release behavior of DEX, GDF-5 and DEX/GDF-5 on the surface-modified samples. An initial burst of DEX, GDF-5 and DEX/GDF-5 release was observed within the first day. Thereafter, the surface-modified samples showed the controlled and sustained release of the coated components. The cumulative percentages were 83.2 ± 7.7%, 48.6 ± 8.3% and 71.9 ± 5.7%/45.2 ± 6.4%, respectively.

### 2.6. In Vitro Cell Proliferation Rate

**Figure 5:**
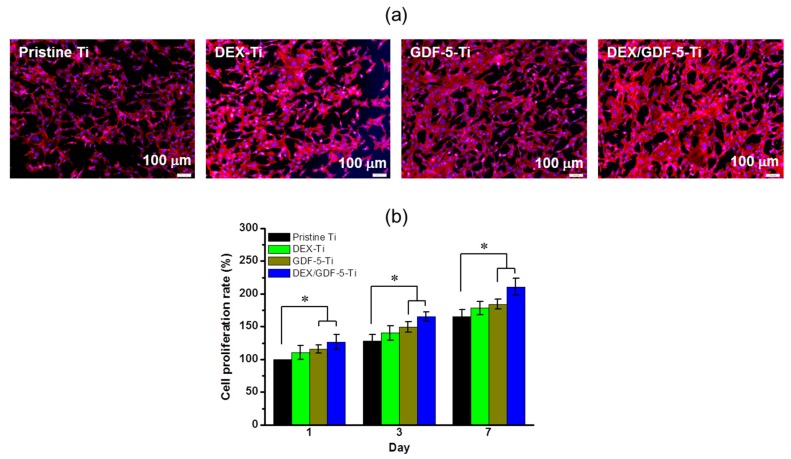
(**a**) Fluorescence images and (**b**) in vitro cell proliferation rates of MC3T3-E1 cells cultured on pristine Ti, DEX–Ti, GDF-5–Ti and DEX/GDF-5–Ti disc samples. The images and rates were examined by fluorescence microscopy at days 14, and cell counting kit-8 (CCK-8) assay at days 1, 3 and 7, respectively. The white bar indicates 100 μm.

The cell proliferation rate and appearance of MC3T3-E1 cells cultured on pristine Ti, DEX–Ti, GDF-5–Ti and DEX/GDF-5–Ti for 1, 3 and 7 days were examined by fluorescence microscopy and cell counting kit-8 (CCK-8) assay, respectively (Figure 5a,b). At day seven, the cells were well proliferated on the surface-modified samples, as compared with pristine Ti. In addition, the cells cultured on DEX/GDF-5–Ti appeared to be actively proliferated. The cell proliferation rate increased on the samples throughout the culture periods. The surface-modified samples showed higher cell proliferation rate than pristine Ti. In particular, the cell proliferation rate was significantly higher on DEX/GDF-5–Ti than pristine, DEX–Ti and GDF-5–Ti. At day seven, the cell proliferation rate on DEX/GDF-5–Ti was 1.28-, 1.18- and 1.14-fold higher than pristine Ti, DEX–Ti and GDF-5–Ti, respectively. The cells seeded on the samples seemed to adhere and proliferate throughout the culture periods. Cells seeded on the surface-modified samples proliferated more actively than cells on pristine Ti. Among the surface-modified samples, DEX/GDF-5–Ti showed the most noticeable cell proliferation.

### 2.7. ALP Activity

**Figure 6:**
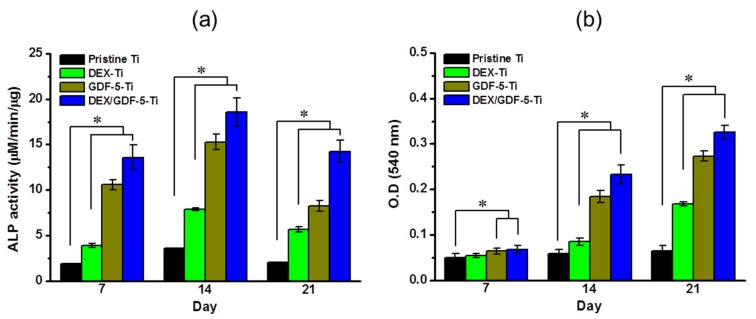
(**a**) Level of (CCK-8) (ALP) activity and (**b**) amount of calcium deposition on pristine Ti, DEX–Ti, GDF-5–Ti and DEX/GDF-5–Ti disc samples cultured with MC3T3-E1 cells for 7, 14 and 21 days. The error bars represent mean ± SD (*n* = 3). These experiments were repeated three times (* *p* < 0.05).

Figure 6a shows the ALP activity of MC3T3-E1 cells cultured on pristine, DEX–Ti, GDF-5–Ti and DEX/GDF-5–Ti samples for 7, 14 and 21 days. ALP activity on the samples increased for 14 days and decreased thereafter. ALP activity was greater on cells cultured on the surface-modified samples throughout the culture periods. Cells cultured on DEX/GDF-5–Ti displayed markedly high ALP activity. At day 14, the ALP activity on DEX/GDF-5–Ti was 5.20-, 2.36- and 1.22-fold larger than that on pristine Ti, DEX–Ti and GDF-5–Ti, respectively.

### 2.8. Calcium Deposition

Calcium deposition on MC3T3-E1 cells cultured on pristine Ti, DEX–Ti, GDF-5–Ti and DEX/GDF-5–Ti samples for 7, 14 and 21 days is expressed in Figure 6b. The samples showed a gradual increase of calcium deposition throughout the culture periods. Similar to ALP activity, increased calcium deposition was observed on the surface-modified samples. In particular, cells cultured on DEX/GDF-5–Ti displayed markedly more calcium deposition. At day 21, the amount of calcium on DEX/GDF-5–Ti was 5.08-, 1.93- and 1.19-fold larger than pristine Ti, DEX–Ti and GDF-5–Ti, respectively.

### 2.9. COL1, ALP and OCN mRNA Expressions

**Figure 7:**
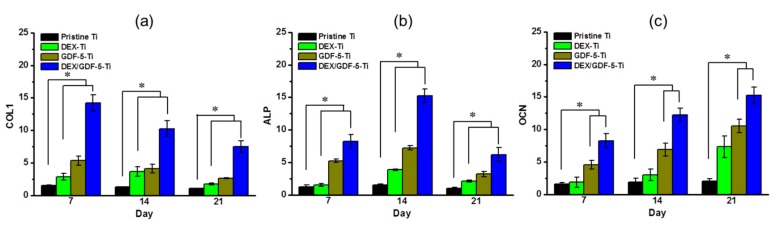
Level of (**a**) type I collagen (COL 1); (**b**) ALP and (**c**) osteocalcin (OCN), mRNA expression for pristine Ti, DEX–Ti, GDF-5–Ti and DEX/GDF-5–Ti disc samples cultured with MC3T3-E1 cells for 7, 14 and 21 days. The error bars represent mean ± SD (*n* = 3). These experiments were repeated three times (* *p* < 0.05).

Figure 7 shows the levels of type I collagen (COL I), ALP and osteocalcin (OCN) mRNA expressions of MC3T3-E1 cells cultured on pristine Ti, DEX–Ti, GDF-5–Ti and DEX/GDF-5–Ti samples for 7, 14 and 21 days. A gradual decrease in COL1 mRNA expression was observed on the samples by 21 days (Figure 7a). The ALP mRNA expression increased for 14 days, and decreased thereafter (Figure 7b). OCN mRNA expression showed a gradual increase for 21 days (Figure 7c). The levels of the mRNA expressions on the surface-modified samples were higher than that on pristine Ti throughout the culture periods. In particular, the highest level was observed on DEX/GDF-5–Ti among the surface-modified samples. At day seven, the level of COL 1 mRNA expression on DEX/GDF-5–Ti was 9.09-, 4.95- and 2.66-fold higher than those on pristine Ti, DEX–Ti and GDF-5–Ti, respectively. At day 14, the level of ALP mRNA expression on DEX/GDF-5–Ti was 9.70-, 3.94- and 2.10-fold higher than those on pristine Ti, DEX–Ti and GDF-5–Ti, respectively. At day 21, the level of OCN mRNA expression on DEX/GDF-5–Ti was 7.10-, 4.32- and 2.85-fold higher than those on pristine Ti, DEX–Ti and GDF-5–Ti, respectively.

### 2.10. Micro-CT Images, Bone Volume (BV), Bone Volume BV/TV, BIC and Removal Torque

**Figure 8:**
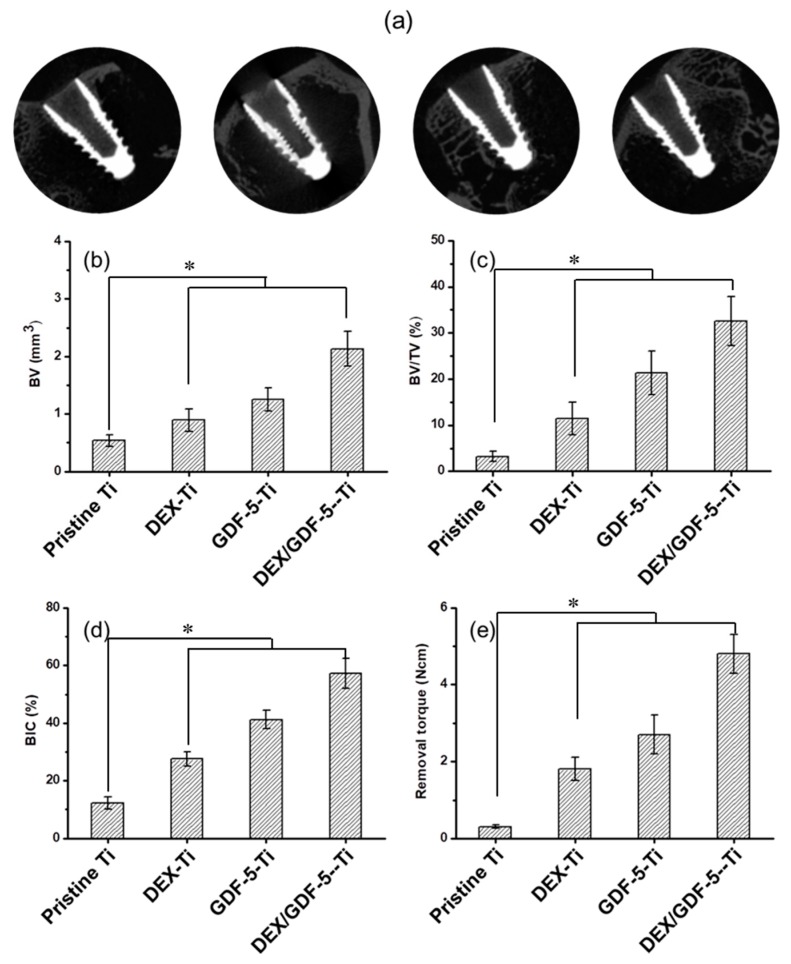
(**a**) Images of micro computed-tomography (micro CT); (**b**) amounts of bone volume (BV); (**c**) percentages of bone volume/tissue volume (BV/TV); (**d**) percentages of bone-implant contact (BIC) and (**e**) values of removal torque on pristine Ti, DEX–Ti, GDF-5–Ti and DEX/GDF-5–Ti screw samples after 4 weeks of implantation. The error bars represent mean ± SD (*n* = 3). These experiments were repeated three times (* *p* < 0.05).

Figure 8a shows the micro-CT images of pristine Ti, DEX–Ti, GDF-5–Ti and DEX/GDF-5–Ti samples implanted into the proximal femur areas of rabbits during implantation for four weeks. Bone volume (BV), bone volume/tissue volume (BV/TV) and bone-implant contact (BIC) were examined using the micro-CT images (Figure 8b–d). BVs at the interfaces between the samples and host bone were 0.5, 0.9, 1.3 and 2.1 mm^3^ in order, respectively (Figure 8b). The percentage of BV/TV on the samples was 3.2, 11.4, 21.3 and 32.6%, respectively (Figure 8c). The percentage of *BIC* on the samples was 12.3, 27.5, 41.2 and 57.2%, respectively (Figure 8d). The removal torque on the samples was 0.3, 1.8, 2.7 and 4.8 N·m, respectively (Figure 8e). These results indicated that new bone was formed at the interfaces between the surface-modified samples and host bone. In particular, the largest amount of bone was produced at the interface between DEX/GDF-5–Ti and host bone. This was attributed to the synergistic effect DEX/GDF-5.

## 3. Discussion

Success of Ti implants after implantation is determined by strong osseointegration with host bone [10]. Osseointegration is deeply correlated with bone formation between Ti implants and host bone. Such osseointegration is particularly influenced by the surface characteristics of the implants including chemical composition, roughness and wettability [3]. To accomplish strong osseointegration, physical, chemical and biological surface modification methods have been reported. To date, widely used Ti implants have been prepared by physical and chemical surface modification methods, however, the surface modifications still remain unsatisfactory. Therefore, we have considered biological surface modification method for improving osseointegration, in which bone-forming drugs or growth factors are coated to the implant surface [11,12].

In this study, we designed and prepared DEX and/or GDF-5 surface-modified Ti implants for improving osseointegration, and evaluated their efficacies on bone formation in vitro and in vivo. DEX is a synthetic glucocorticoid that has been used clinically as an anti-inflammatory drug. The long-term administration of DEX may cause or exacerbate osteoporosis [13]. So, short-term administration is a requisite for improving bone formation. In addition, the concentration of DEX affects the differentiation of human osteoblastic cells [14]. Kouji et al. reported that concentrations ranging from 10^−8^ to 10^−6^ M enhanced the osteoblastic differentiation [14]. Despite the osteogenic capacity of DEX, it has low bioavailability because of its poor water solubility. β-CD can improve the bioavailability of DEX, because DEX can form an inclusion complex with the cavity of β-CD in a 1:1 molar ratio [15]. The surface modification of Ti implants with β-CD helps anchor DEX on the surface as part of the inclusion complex. GDF-5, a member of the bone morphogenetic proteins family, is a growth factor for the enhancement of bone formation. GDF-5 plays a leading role in regenerating bone on damaged periodontal ligament tissue [6]. However, GDF-5 is expensive and a high dose can be required to improve bone formation. The need for a high dose is attributed to the short half-life of GDF-5 due to its degradation through proteolytic cleavage [16]. Heparin is a glycosaminoglycan that can bind with GDF-5 [8], facilitating surface-coating of GDF-5 onto heparin-conjugated Ti implants. Therefore, we prepared β-CD-conjugated heparin for coating DEX and GDF-5 onto Ti implants, confirmed by confirmed by ^1^H-NMR and XPS analyses (Figure 1 and Figure 2).

The surface characteristics of Ti including morphology and wettability affect osteogenic activity at the interface between the material and host bone [17]. Surface roughness and wettability of Ti samples can modulate the response of cells [17]. For example, Ti samples with the combination characteristics of micro/nanostructures and hydrophilicity showed osteoblast differentiation in vitro and osseointegration in vivo [3,18]. Surface modification of Ti disc samples using DEX and/or GDF-5 produced rougher and more hydrophilic surfaces compared with pristine Ti, as shown using SEM and contact angle measurements (Figure 3 and Table 1).

Controlled release of bone-forming drugs or growth factors in a sustained manner plays a significant role in improving bone formation between implants and host bone. Some methods, including uses of β-CD, heparin, click reaction, and so forth, have been reported for the controlled releases [12,19]. Fiorica et al., reported that β-CD-conjugated hyaluronic acid hydrogel allows for the controlled and sustained release of DEX [19]. Sustained 28-day release of GDF-5 on GDF-5-coated Ti discs has been described by Yang and co-workers [12]. The releases of DEX and/or GDF-5 was controlled by using CD–Hep and the surface-modified samples showed the controlled releases for 28 days in a sustained manner (Figure 4).

In vitro evaluations of cell proliferation and bone formation on Ti implants are generally done by examining cell proliferation rate, ALP activity, calcium deposition and expression of bone-related mRNA genes. ALP activity is a marker of the osteoblastic phenotype, and is suited as a measurement of bone formation [20]. Calcium deposition is an important phenomenon that occurs during the process of mineralization [21]. COL1, ALP and OCN are representative proteins translated from mRNA during proliferation, bone maturation and bone mineralization, respectively [22,23,24]. Some researchers found that DEX or GDF-5 surface modification improve the proliferation, ALP activity, calcium deposition and the expression of bone-related mRNA genes of osteoblast cells cultured on the surface-modified implants [14,25]. As expected, MC3T3-E1 cells cultured on DEX and/or GDF-5 surface-modified Ti implants exhibited improved cell proliferation, ALP activity, calcium deposition and expression of bone-related mRNA genes including COL1, ALP and OCN. In particular, DEX/GDF-5–Ti sample showed superior osteogenic activity, which may attribute to the roles of DEX and GDF-5 (Figure 5, Figure 6, Figure 7 and Figure 8).

**Figure 9:**
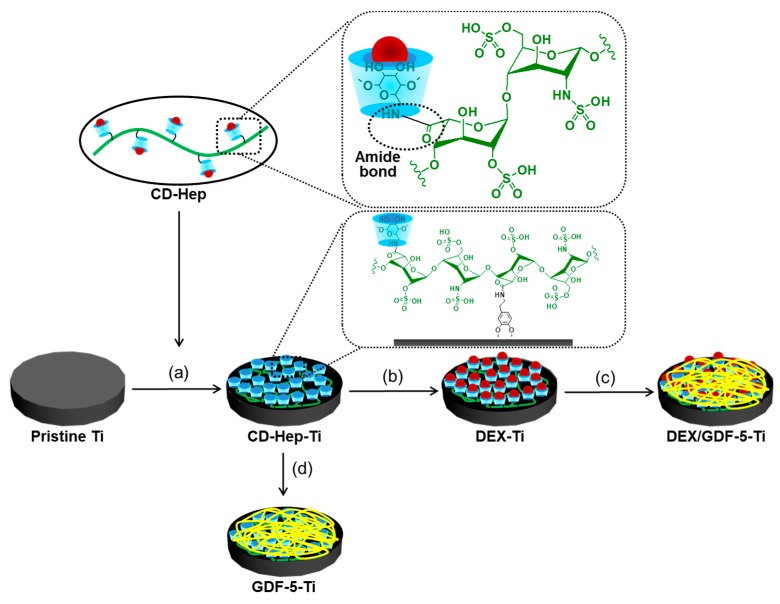
Schematic illustration on the surface modification of titanium discs (diameter: 8 mm; height: 1 mm) and screws (φ 3.5 × 8.5 mm) using DEX and/or GDF-5 by β-CD and heparin. The two types were surface-modified by same process.

In this study, micro-CT was carried out to evaluate how well the surface modifications enhanced bone formation and osseointegration [3,24]. DEX and/or GDF-5 surface-modified Ti samples showed improved bone formation between the implants and host bone. Among the surface-modified samples, DEX/GDF-5–Ti produced the highest level of bone volume (Figure 9). The effect of DEX and/or GDF-5 surface-modified Ti implants on the enhancement of bone formation in vivo has been suggested to be resulted from the anti-inflammation effect of DEX and the osteogenic activity of GDF-5. There have been many studies on the effect of DEX on osteoblast and human bone-derived cells [14]. One study reported that DEX enhanced ALP activity on rat osteosarcoma cells [14]. For the expression of OCN mRNA expression, DEX increased in rat osteoblasts [14]. Furthermore, DEX induced osteoblastic differentiation in bone marrow stromal cells [14]. DEX also exhibited positive stimulation on the differentiation of osteoprogenitor cells and immature osteoblasts [14]. Some studies indicated that GDF-5 is essential for bone formation [25]. For example, it was found that GDF-5 induces osteogenesis and angiogenesis in vitro and in vivo [25]. In a murine calvarial model, GDF-5, together with human COL 1, formed new bone by endochondral ossification [25]. In addition, GDF-5 signaling enhanced the expression of pre-osteoblast and secretory osteoblast-related genes such ALP [25]. From these facts, we suggested that surface modification of Ti implant using a combination of DEX and GDF-5 might induce active bone remodeling at the interface between the surface-modified implant and host bone by a continuous process of the ossification by osteoblasts and bone resorption by osteoclasts. Consequentially, the combination of DEX and GDF-5 can be used as one of surface modifications for improving the osseointegration of Ti implants.

## 4. Experimental Section

### 4.1. Materials

Samples of Ti disc (8 mm diameter, 1 mm height) and screw (φ 3.5 × 8.5 mm) with a smooth surface were obtained from DIO Co. (Busan, Korea). β-CD–NH_2_, dopamine hydrochloride (DOPA) and DEX, β-glycero phosphate disodium salt hydrate, ascorbic acid and DEX were provided by Sigma-Aldrich (St. Louis, MO, USA). Heparin (12,000–15,000 g/mol) was purchased from Acros Organics/Thermo Fisher (Waltham, MA, USA). Dulbecco’s Modified Eagle Medium (DMEM), fetal bovine serum (FBS), phosphate buffered saline (PBS) and penicillin-streptomycin (PS) solutions were purchased from Gibco BRL (Gaithersburg, MD, USA). 4-(4,6-Dimethoxy-1,3,5-triazin-2-yl)-4-methylmorpholinium chloride (DMT-MM) was purchased from Wako (Osaka, Japan). Dialysis tube (cut-off: 25 kDa) was used for the purification of CD–Hep. GDF-5 and its enzyme-linked immunosorbent assay (ELISA) kit were purchased from Peprotech Inc. (Rocky Hill, NJ, USA). MC3T3-E1 osteoblast precursor cell line was supplied by the Korean Cell Line Bank (Seoul, Korea). All organic solvents were used as received without any additional purification.

### 4.2. Preparation of DEX–Ti, GDF-5–Ti and DEX/GDF-5–Ti of Disc and Screw Types

Figure 9 shows the schematic illustration for preparation of DEX–Ti, GDF-5–Ti and DEX/GDF-5–Ti. Surface-modified Ti disc samples were used for XPS, SEM, contact angle measurement and in vitro tests, including the determination of the proliferation rate of MC3T3-E1 cells, ALP activity, calcium deposition and expressions of COL I, ALP and OCN mRNA. Screw type samples were used for in vivo tests including micro-CT and removal torque value.

#### 4.2.1. Preparation of CD–Hep

Heparin (0.07 mmol, 1 g) was dissolved in distilled water (50 mL) and continuously stirred at room temperature for 1 h to activate the carboxyl group of heparin. β-CD–NH_2_ (1.75 mmol, 2 g) was added to the heparin solution and reacted at room temperature for 24 h. The reactant was dialyzed using a dialysis tube (5 kDa cut-off). After filtering, the purified solution was lyophilised at −90 °C and stored in a desiccator with a nitrogen gas supply before use. Formation of CD–Hep was characterized by ^1^H-NMR (Bruker Avance 400; Bruker Corp., Billerica, MA, USA) spectroscopy, measured in D_2_O.

#### 4.2.2. Preparation of DEX–Ti

To a mixture of CD–Hep (10 mmol, 1.1 g) and DMT-MM (1 mmol, 27 mg) in 10 mM Tris-buffer (pH 4.5, 10 mL), DOPA (2 mg/mL) was added and reacted at room temperature for 1 day [26]. The product was washed three times with distilled water. DEX (0.1 mmol, 39 mg) dissolved in methanol (2 mL) was added to a mixture of CD–Hep surface-modified Ti sample in distilled water (50 mL). The mixture was continuously stirred at room temperature until the complete evaporation of the organic solvent. After filtering, each sample was washed three times with methanol, followed by three distilled water washes. The final product (DEX–Ti) was dried with a continuous flow of nitrogen gas and stored in a desiccator supplied with nitrogen gas before use.

#### 4.2.3. Preparation of GDF-5–Ti

Heparin (0.07 mmol, 1 g) was added to a mixture of Ti–COOH samples in distilled water (50 mL). After filtering, the samples were washed three times with distilled water and dried with a continuous flow of nitrogen gas. Heparin coated samples were added to an aqueous GDF-5 solution (50 ng/40 mL, PBS pH 7.4) and continuously shaken at 4 °C for 24 h. After washing three times with distilled water, the final samples were lyophilized at −90 °C and stored in a freezer (−20 °C) before use.

#### 4.2.4. Preparation of DEX/GDF-5–Ti

DEX/GDF-5–Ti samples were prepared according to Section 4.2.2 and Section 4.2.3 in order.

### 4.3. XPS

The surface element compositions (Ti2p, C1s, N1s and O1s) of pristine and surface-modified Ti samples were examined by using a K-Alpha XPS system (Thermo Electron Corp., Waltham, MA, USA) with a 10 kV source and monochromated X-ray beam (photoelectron energy 1486.6 eV). Wide-scan XPS and high-resolution examinations were done at a pass energy of 187.8 and 20 eV, respectively. Wide XPS scans were recorded from 100 and 800 eV at a grazing angle of 90° in a high vacuum (<3.1 × 10^−9^ Torr).

### 4.4. SEM

Pristine and surface-modified Ti samples were attached to a metal mount using carbon tape and gold-coated using an IB-3 ion sputter-coater (Eiko Engineering Co., Ltd., Hitachinaka, Japan). The surface morphologies were observed by SEM (S-2300; Hitachi, Tokyo, Japan) at 15 kV, as observed at a magnification of 5 k.

### 4.5. Contact Angle Measurement

Water (20 µL) water was dropped on the surfaces of pristine and surface-modified Ti samples. The water contact angles of the samples were measured using a model G-1 contact angle meter (Erma, Tokyo, Japan).

### 4.6. Coating Efficiency of DEX, GDF-5 and DEX/GDF-5 on DEX–Ti, GDF-5–Ti and DEX/GDF-5–Ti, and Their Release Behaviors

After the coating processes as explained in Section 4.2, the remaining solution was analyzed by high performance liquid chromatography (HPLC) and a microplate reader (Bio-Rad, Hercules, CA, USA) using an enzyme-linked immunosorbent assay (ELISA) to calculate the coating efficiency of DEX and GDF-5. For in vitro release tests of DEX and GDF-5, DEX–Ti, GDF-5–Ti and DEX/GDF-5–Ti samples immersed in vials filled with 3 mM PBS were incubated at 37 °C and 100 rpm. At 1, 6, and 12 h, and 1, 3, 5, 7, 14, 21, and 28 days, the PBS solution was collected and 3 mL fresh PBS was added to the vials. The collected solutions were measured by HPLC and a microplate reader using ELISA at 450 nm to determine the release of DEX and GDF-5.

### 4.7. Culture of MC3T3-E1 Cells

MC3T3-E1 cells were cultured in 48-well plates with culture medium composed of 200 µL DMEM containing 10% FBS and 1% PS in a humidified incubator set at 37 °C and 5% CO_2_. The culture medium was replaced daily for 3 days during the culture. A fixed number of cells (5 × 10^3^ cells/well) was used to evaluate cell proliferation rate, ALP activity, calcium deposition, and expression of mRNA for COL I, ALP and OCN.

### 4.8. In Vitro MC3T3-E1 Cell Proliferation Assay

A fixed number of cells (5 × 10^3^ cells/well) were seeded on pristine and surface-modified Ti samples and cultured with culture medium for 1, 3 and 7 days. At each predetermined time interval, the cell appearance and rate of cell proliferation were examined by fluorescence microscopy using a model DP2-BSW microscope (Olympus, Tokyo, Japan) and CCK-8 assay, respectively. For the observation of the appearance, the nuclei and cytoskeletons of cells cultured on the samples were stained by 4′-6-diamidino-2-phenylindole (DAPI) and F-actin method using rhodamine phalloidin (Invitrogen, Carlsbad, CA, USA) and observed at ×200 magnification. For the measurement of the cell proliferation rate, CCK-8 reagent was added to the samples and incubated for 3 h. After shaking for additional 10 min, the supernatants were measured using a microplate reader at 450 nm.

### 4.9. ALP Activity and Calcium Deposition Assays

Osteogenic medium containing 10% FBS, 1% PS, 10 mM β-glycero phosphate disodium salt hydrate, 300 µM ascorbic acid and 0.1 µM dexamethasone was used for the ALP activity and calcium deposition assays of MC3T3-E1 cells (initial seeding amount: 1 × 10^5^ cells/well) cultured on pristine Ti, DEX–Ti, GDF-5 and DEX/GDF-5–Ti for 7, 14 and 21 days. To assay ALP activity, radioimmunoprecipitation assay buffer (RIPA buffer), 50 mM Tri-HCl pH 7.4, 150 mM NaCl, 0.25% deoxycholic acid, 1% Nonidet P-40 (NP-40) and 1 mM ethylenediaminetetraacetic acid (EDTA) containing protease inhibitor cocktail (Roche, Mannheim, Germany) was added to the PBS (pH 7.4) rinsed samples at the predetermined time intervals. The cells were lysed in RIPA buffer for 20 min on ice and the lysates were centrifuged at 4 °C for 10 min to remove cell debris. *p*-Nitrophenyl phosphate (PNPP) was added to the supernatants and incubated. NaOH (1 N, 50 µL) was used to terminate the reaction with PNPP. ALP activity was determined by measuring the conversion of PNPP to *p*-nitrophenol using a microplate reader at 410 nm.

For the calcium deposition assay, PBS-rinsed samples were fixed with 3.7% formaldehyde for 20 min and rinsed again. According to an alizarin red S (ARS) stain protocol, the samples were treated with 40 mM ARS solution and incubated at 37 °C for 1 h in an atmosphere of 50% CO_2_. The extracted samples were rinsed three times with distilled water. The stained cells were desorbed by treatment of hexadecylpyridinium chloride (10%). The amount of calcium deposited on the samples was measured using a microplate reader at 540 nm.

### 4.10. Real-Time Polymerase Chain Reaction (qPCR) Analysis

Total RNA of MC3T3-E1 cells cultured on Ti samples for 7, 14 and 21 days was isolated using an RNeasy Plus Mini Kit (Qiagen, Hilden, Germany, USA). The extracted RNA (1 µg) was transcribed into cDNA with AccuPower CycleScript RT Premix (Bioneer, Daejeon, Korea). The qPCR amplifications were carried out using AccuPower PCR PreMix (Bioneer). The qPCR products were detected using iQ SYBR Green supermix (Bio-Rad). The values of threshold cycle were determined by a comparative CT method. At each analysis time, the fold-change of the control condition was set as one-fold. Table 1 shows the primers used for this study. The qPCR amplifications were carried out under a cycle of 10 s at 95 °C, 30 s at 57–62 °C (COL1: 60 °C, ALP: 61 °C, OCN: 57 °C) and 30 s at 72 °C for 45 cycles after the initial denaturation step for 10 min at 95 °C.

### 4.11. In Vivo Animal Test

The protocols for animal test was approved by the Institutional Animal Care and Use Committee of Kyung Hee University (KHUASP(SE)-17-006). Ten white New Zealand rabbits (2.5–3.5 kg; D.Y. Biotech., Seoul, Korea) were used. The animals were anesthetized using Ketara and Rompun, disinfected using Betadine, and their femur and proximal tibia were shaven for the surgery. Lignospan standard (0.5 mL) was injected into the subcutaneous tissues and cut using a surgical scalpel. Five screws for micro-CT and histological analyses and five screws for removal torque value measurement for each Ti sample were implanted into the femur and the proximal tibia, respectively. Periosteum and fascia, and skin were sutured with Ethicon and Ethicon Ethilon, respectively. Cefamandole for 3 days was used for preventing further complications after the surgery. After 4 weeks, the tissues around the implanted sites were harvested after sacrificing the animals with KCl. The sites were evaluated by micro-CT and removal torque measurements.

### 4.12. Micro-CT Images

Micro-CT (SkyScan 1076; Bruker, Brussels, Belgium) was used for the examination of bone volume (BV), bone volume/tissue volume (BV/TV) and bone-implant contact (BIC) between the Ti samples and host bone. Measurement parameters were: X-ray source voltage of 100 kV; X-ray source current of 100 µA; 0.6° angular increments over 360°, and; exposure for 460 ms. A distance of 1.4 mm from longitudinal axis of the samples was selected as the region of interest. Skyscan CT-analyzer software (Bruker) was used for the analysis of the scanned images. For calibration of the acquired images, the CT number of air and water were set as −1000 and 0 Hounsfield units, respectively.

### 4.13. Removal Torque Measurement

Removal torques between Ti samples and host bone were measured using a model 100R computerized material testing machine (Admet, Norwood, MA, USA). The sample-implanted tissues were fixed on mounts and turned with the machine at a speed of 0.4°/s. The removal torque was determined by the maximum torque.

### 4.14. Statistical Analyses

Statistical analyses were performed by one-way analysis of variance (ANOVA) using SPSS software (SPSS Inc., Chicago, IL, USA) and expressed as the mean ± standard deviation. A *p*-value * < 0.05 was considered statistically significant. MedCalc statistical software (MedCalc Software bvba, Ostend, Belgium) using α (*p* = 0.05) and power (1 − β = 0.8) was used for the calculation of the number of rabbits used for this study.

## 5. Conclusions

We prepared DEX and/or GDF-5 surface-modified Ti samples of disc and screw types, and evaluated their efficacies on the improvement of bone formation and osseointegration in vitro and in vivo. DEX/GDF-5 surface-modified Ti showed enhanced bone formation and osseointegration, along with roughness, wettability and controlled release. Consequently, we suggested that the surface modification using DEX/GDF-5 may a good method for clinical use for improved dental or orthopedic implants.

## Figures and Tables

**Table 1:** Percentages of Ti2p, C1s, N1s, O1s and S2p on pristine Ti, DEX–Ti, GDF-5–Ti and DEX/GDF-5–Ti, and their surface contact angles. XPS signals: Ti2p (461.6 eV); O1s (530.8 eV); S2p (162.3 eV); C1s (285.9 eV).

Samples	Ti2p (%)	C1s (%)	N1s (%)	O1s (%)	S2p (%)	Contact Angle (°)
Pristine Ti	22.54	13.12	-	64.34	-	78
DEX–Ti	18.54	17.21	4.53	58.38	1.34	54
GDF-5–Ti	17.87	22.79	3.23	56.11	-	47
DEX/GDF-5–Ti	15.22	24.42	2.76	57.60	-	50
